# Protective LRRK2 R1398H Variant Enhances GTPase and Wnt Signaling Activity

**DOI:** 10.3389/fnmol.2016.00018

**Published:** 2016-03-08

**Authors:** Jonathon Nixon-Abell, Daniel C. Berwick, Simone Grannó, Victoria A. Spain, Craig Blackstone, Kirsten Harvey

**Affiliations:** ^1^Department of Pharmacology, UCL School of Pharmacy, University College LondonLondon, UK; ^2^Neurogenetics Branch, National Institute of Neurological Disorders and Stroke – National Institutes of Health, BethesdaMD, USA; ^3^Department of Life, Health and Chemical Sciences, The Open UniversityMilton Keynes, UK

**Keywords:** GTPase activity, LRRK2, Parkinson’s disease, protective genetic variant, Wnt signaling

## Abstract

Mutations in *LRRK2* are a common cause of familial and idiopathic Parkinson’s disease (PD). Recently, the LRRK2 GTPase domain R1398H variant was suggested in genetic studies to confer protection against PD but mechanistic data supporting this is lacking. Here, we present evidence that R1398H affects GTPase function, axon outgrowth, and Wnt signaling in a manner opposite to pathogenic *LRRK2* mutations. LRRK2 R1398H GTPase domain dimerization and GTP hydrolysis were increased whereas GTP binding was reduced, leading to a decrease in active GTP-bound LRRK2. This protective variant also increased axon length of primary cortical neurones in comparison to wild-type LRRK2, whereas the R1441G LRRK2 pathogenic mutant decreased axon outgrowth. Importantly, R1398H enhanced the stimulatory effect of LRRK2 on canonical Wnt signaling whereas the G2385R risk variant, in accordance with all previously tested pathogenic LRRK2 mutants, had the opposite effect. Molecular modeling placed R1398H in close proximity to PD-causing mutations suggesting that this protective LRRK2 variant, like familial mutations, affects intramolecular RocCOR domain interactions. Thus, our data suggest that R1398H LRRK2 is a *bona fide* protective variant. The opposite effects of protective versus PD associated *LRRK2* variants on GTPase function and canonical Wnt signaling activity also suggests that regulation of these two basic signaling mechanisms is important for neuronal function. We conclude that LRRK2 mediated Wnt signaling and GTPase function are fundamental in conferring disease susceptibility and have clear implications for therapeutic target identification.

## Introduction

Autosomal-dominant mutations in *LRRK2*, encoding leucine-rich repeat kinase 2 (LRRK2), are the most common known cause of inherited Parkinson’s disease (PD; Paisán-Ruíz et al., 2004; Zimprich et al., 2004). Patients with *LRRK2* mutations display symptoms and brain pathologies that are largely indistinguishable from those of individuals with idiopathic PD (Paisán-Ruíz et al., 2004; Zimprich et al., 2004; Ross et al., 2006; Kumari and Tan, 2009). Thus, determining the biological role of LRRK2 is of paramount importance to understanding the etiology of PD, and likely to help uncover new therapeutic strategies.

**FIGURE 1:**
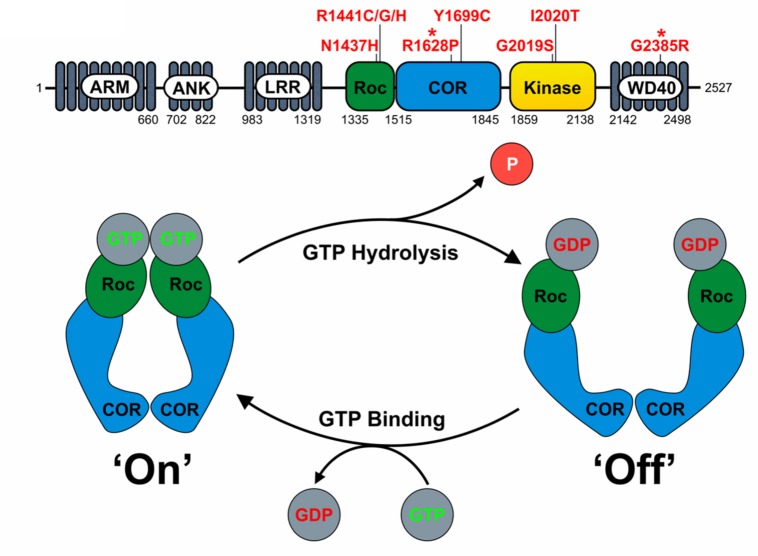
**LRRK2 domain structure and proposed function as a GAD GTPase. (A)** The domain structure of LRRK2 and location of key pathogenic mutations and PD risk variants (^∗^) is depicted. Note the central catalytic core consisting of the RocCOR tandem and kinase domains. **(B)** The assumed behavior of the LRRK2 RocCOR tandem as a GAD protein is represented graphically. In this model, COR domains are a constitutive dimerization device, whilst Roc domains cycle between a GDP-bound and monomeric ‘off’ state, and a GTP-bound and dimeric ‘on’ state.

LRRK2 is a multifunctional protein containing both kinase and GTPase activities and a number of protein–protein interaction domains (**Figure 1**). The ‘catalytic core’ is contained within the Roc (Ras of complex proteins), COR (C-terminal of Roc) and kinase domains (**Figure 1**) and appears essential for LRRK2 function (Berwick and Harvey, 2011). As the only hereditary mutations that are proven to cause PD fall within exons coding for the Roc, COR and kinase domains, the effects of pathogenic mutations on LRRK2 enzymatic activities require further investigation.

The precise extent of the LRRK2 GTPase domain remains controversial. Restricting the LRRK2 GTPase domain to the Roc domain can be justified by the similarity to Ras family small GTPases. Arguing against this are, the observations that isolated Roc protein appears to hydrolyse GTP at a greatly reduced rate compared to full-length LRRK2 (Gilsbach and Kortholt, 2014) and that throughout evolution, Roc and COR domains never occur without each other (Bosgraaf and van Haastert, 2003). This supports the definition of a LRRK2 GTPase “RocCOR tandem” domain.

Based on homology to *C. tepidum* Roco protein, the LRRK2 RocCOR tandem is predicted to fall into the GAD (G proteins activated by nucleotide-dependent dimerization) class of molecular switches (Gotthardt et al., 2008; Gasper et al., 2009; Gilsbach and Kortholt, 2014). Under the GAD model, two LRRK2 molecules are expected to dimerize via a constitutive interaction between the COR domains holding the two Roc domains in close proximity (**Figures 1** and **3A,B**). Binding of GTP to the Roc domains results in protein dimerization allowing binding of effector proteins. GTP hydrolysis disrupts Roc dimerization, leading to the dissociation of effector proteins (Gotthardt et al., 2008; Gasper et al., 2009; Gilsbach and Kortholt, 2014).

All known PD-causing mutations located within the LRRK2 RocCOR domain have been reported to increase GTP-binding, decrease GTPase activity, or both (**Table 1**) supporting the idea that shifting LRRK2 to the GTP-bound ‘on’-state promotes neurodegeneration.

**Table 1:** The effect of PD-causing mutations in the LRRK2 RocCOR tandem domain on GTP-binding and GTPase activity.

Mutation	LRRK2 construct	GTP binding	GTP hydrolysis	Prediction	Reference
N1437H	Full length	↑	ns	↑ GTP-bound	Aasly et al., 2010
R1441C	Full length	↑	ns	↑ GTP-bound	West et al., 2007
R1441C	Full length	—	↓	↑ GTP-bound	Li et al., 2007
R1441C	Full length	—	↓	↑ GTP-bound	Lewis et al., 2007
R1441C	Roc	ns	↓	↑ GTP-bound	Deng et al., 2008
R1441C	Full length	ns	↓	↑ GTP-bound	Xiong et al., 2010
R1441C	Roc, COR, kinase	—	—	No change	Xiong et al., 2010
R1441C	970-2527	ns	↓	↑ GTP-bound	Pungaliya et al., 2010
R1441C	Full length	↑	ns	↑ GTP-bound	Stafa et al., 2012
R1441G	Full length	↑	ns	↑ GTP-bound	West et al., 2007
R1441G	Full length	—	↓	↑ GTP-bound	Li et al., 2007
R1441G	Full length	ns	↓	↑ GTP-bound	Xiong et al., 2010
R1441H	Roc	↑	↓	↑ GTP-bound	Liao et al., 2014
“R1441”A	*C. tepidum* RocCOR	—	↓	↑ GTP-bound	Gotthardt et al., 2008
Y1699C	Full length	↑	ns	↑ GTP-bound	West et al., 2007
Y1699C	Full length	ns	↓	↑ GTP-bound	Xiong et al., 2010
Y1699C	Full length	—	↓	↑ GTP-bound	Daniëls et al., 2011
Y1699C	Full length	↑	ns	↑ GTP-bound	Stafa et al., 2012
“Y1699”C	*C. tepidum* RocCOR	—	↓	↑ GTP-bound	Gotthardt et al., 2008

*Based on published data, the pathogenic N1437H, R1441C, R1441G, R1441H, and Y1699C mutations all elicit an increase in the fraction of GTP-bound LRRK2. Only one construct, an R1441C RocCOR-kinase, displayed no changes in GTP-binding or GTP hydrolysis; however, this mutation decreased GTPase activity in full length LRRK2 in the same publication. Note that mutations at residues equivalent to R1441 and Y1699 in C. tepidum Roco protein produce consistent results. Key: up and down arrows represent increases and decreases, respectively; dashes represent no change; ‘ns’ indicates ‘not studied.’*

A consensus on the cellular role of LRRK2 is still lacking, with numerous competing – though not mutually exclusive – functions reported. Nonetheless, a comprehensive literature review identified cell biological processes involving LRRK2 that appear to be reproducible (Berwick and Harvey, 2013) including effects on membrane trafficking (Piccoli et al., 2011; Ramonet et al., 2011; reviewed by Gómez-Suaga et al., 2014), cytoskeletal function (Parisiadou et al., 2009; Law et al., 2014; reviewed by Gómez-Suaga et al., 2014) and signal transduction pathways including MAPK, Wnt, TLR, and NFAT pathways (Berwick and Harvey, 2011; Boon et al., 2014; reviewed by Gómez-Suaga et al., 2014).

A growing body of data supports the importance of deregulated canonical Wnt signaling in neurodegenerative disease pathogenesis, including PD (Berwick and Harvey, 2014; Inestrosa and Varela-Nallar, 2014). At least six proteins implicated in PD have been described to modulate this pathway, whilst development of the midbrain dopaminergic neurones that are typically lost in PD is acutely dependent on canonical Wnt signaling (Berwick and Harvey, 2014). Furthermore, decreased Wnt signaling has been reported in PD patients (Cantuti-Castelvetri et al., 2007), as well as in various animal models of parkinsonism (L’Episcopo et al., 2011; Gollamudi et al., 2012). Since Wnt ligands are well established as neuroprotective (Berwick and Harvey, 2014; Inestrosa and Varela-Nallar, 2014), the idea that decreased canonical Wnt signaling is involved in PD pathogenesis is an attractive hypothesis. Importantly, evidence for a crucial role of LRRK2 in canonical Wnt signaling is accumulating. Protein–protein interactions between LRRK2 and a number of Wnt signaling components have been reported, including interactions with disheveled (DVL) proteins, the serine/threonine kinase GSK3β, and LRP6, a Wnt signaling transmembrane receptor (Sancho et al., 2009; Lin et al., 2010; Berwick and Harvey, 2012). Furthermore, over-expressed wild-type LRRK2 enhances the signal strength of activated canonical Wnt signaling (Berwick and Harvey, 2012). Intriguingly, this effect of LRRK2 is weakened by PD-causing mutations in three distinct catalytic domains of LRRK2 – R1441C in the Roc domain, Y1699C in the COR domain, and G2019S in the kinase domain (Berwick and Harvey, 2012) – suggesting that impaired Wnt signaling is a common pathogenic mechanism of familial *LRRK2* mutations.

Recently, a number of genetic screens have reported an inherited R1398H *LRRK2* variant in the Roc domain (Chen et al., 2010; Tan et al., 2010; Ross et al., 2011; Heckman et al., 2013, 2014) that appears to confer decreased risk of PD. This could prove extremely informative, since a protective variant can be expected to display the opposite behavior to pathogenic variants in disease-relevant assays. In a single study, R1398H was reported to display decreased kinase activity compared to wild-type LRRK2 (Tan et al., 2010). However, this observation should be treated with caution, as this study also found the G2385R risk variant to have increased kinase activity, in contrast to other reports (Jaleel et al., 2007; West et al., 2007; Nichols et al., 2010; Rudenko et al., 2012). In any case, the need to elucidate the functional relevance of the R1398H experimentally is clear.

Here, we report the behavior of the LRRK2 R1398H variant in five assays for which the effect of a *bona fide* protective mutation in the LRRK2 Roc domain can be predicted: LRRK2 RocCOR tandem domain dimerization, LRRK2 GTP-binding, LRRK2 GTPase assays, axon outgrowth, and canonical Wnt signaling assays. Remarkably, R1398H displays the opposite behavior to pathogenic mutants in all experiments. Furthermore, molecular modeling studies suggest that this amino acid substitution is likely to affect intramolecular RocCOR interactions, consistent with the predicted mode of action for PD-causing mutations in the RocCOR tandem. Thus our data (1) provide strong experimental support for the status of LRRK2 R1398H as a genuine protective variant; (2) increase the weight of evidence that GTP-bound LRRK2 is pathogenic; and (3) provide further data indicating that decreased canonical Wnt signaling is a key pathomechanism underlying PD.

## Materials and Methods

### Molecular Cloning

pDS-BAIT (pDS; Dualsystems Biotech) plasmids containing the LRRK2 Roc and RocCOR domains (encoding amino acids 1330–1515 and 1335–1845, respectively), pACT2 (Clontech) containing the LRRK2 RocCOR domain and pYTH16 containing the intracellular domain of LRP6 (amino acids 1416–1613) have been described previously (Daniëls et al., 2011; Berwick and Harvey, 2012). pCHMWS vectors expressing 3× FLAG-tagged wild-type LRRK2 and LRRK2-T1348N were a generous gift from Dr. Jean-Marc Taymans (Daniëls et al., 2011). pRK5 myc-LRRK2 has also been described previously (Sancho et al., 2009). R1398H, R1398H/R1441G and G2385R mutations were introduced using the QuikChange Lightening site-directed mutagenesis kit (Agilent) according to the manufacturer’s instructions. All constructs were verified by DNA sequencing.

### Culture of Immortalized Cell Lines

HEK293 cells and SH-SY5Y cells were grown in Dulbecco’s modified Eagle’s medium (DMEM) supplemented with 10% (v/v) fetal bovine serum, 100 U/ml penicillin G and 100 μg/ml streptomycin at 37°C and 5% CO_2_. Transient transfection was performed using FuGENE HD (Roche) according to the manufacturer’s instructions, using a 2.5 μL transfection reagent to 1 μg DNA ratio. In all cases, cells were harvested 24 h after transfection.

### Quantitative Yeast-Two Hybrid

The L40 yeast strain (Invitrogen) was co-transformed with pDS Roc or RocCOR bait and pACT2 wild-type or mutant RocCOR prey constructs, and the Y190 yeast strain (Clontech) was co-transformed with the pYTH16 LRP6 intracellular domain bait and pACT2 wild-type or mutant RocCOR prey constructs. Transformations were spread on selective dropout media (Clontech) lacking leucine and tryptophan for transformation controls, or leucine, tryptophan and histidine, supplemented with 0.5 mM 3-aminotriazole (L40 strain) or 10 mM 3-aminotriazole (Y190 strain; for suppression of ‘leaky’ histidine expression; Sigma-Aldrich) for nutritional selection. After incubation at 30°C for 3 days, prototrophic colonies were picked and used to inoculate minimal SD (Clontech) media lacking leucine and tryptophan. Samples were subsequently incubated shaking at 30°C overnight. Cell pellets were then resuspended in Z-buffer (60 mM Na_2_HPO_4_, 40 mM NaH_2_PO_4_, 10 mM KCl, 1 mM MgSO_4_.7H_2_O) containing 40 mM β-mercaptoethanol, followed by lysis in 0.1% (w/v) SDS (Sigma-Aldrich) and 0.1% (v/v) chloroform (Sigma-Aldrich). After the addition of chloropheno-red-β-D-galactopyranoside (Sigma-Aldrich), the color change was recorded at 540 nm and readings adjusted for turbidity of the yeast suspension at 620 nm. The background signal (bait plus empty pACT2 vector) was subtracted from each reading and values were normalized to the wild-type RocCOR response, which was set at 100%. All protein interactions were assayed in three to five independent experiments in triplicate.

### Molecular Modeling

Molecular modeling was performed on the *C. tepidum* Roco structure (PDB: 3DPU; Gotthardt et al., 2008) using Chimera (Pettersen et al., 2004). Amino acid substitutions were performed with the *swapaa* command using the Dunbrack backbone-dependent rotamer library (Dunbrack, 2002).

### GTP-Binding Assay

HEK293 cells were transfected with 3× FLAG-tagged T1348N, R1398H or wild-type LRRK2. The GTP binding assay was performed similarly as described by others (Korr et al., 2006). Briefly, cells were lysed for 10 min on ice in lysis buffer G (100 mM Tris/HCl pH 7.5, 50 mM KCl, 1 mM EDTA, 0.1 mM DTT, 5 mM MgCl_2_, 1% Triton X-100, protease inhibitor cocktail, Roche), and lysates were centrifuged for 10 min at 20,000 *g* and 4°C. Supernatants containing 100 μg protein each, as assessed by QuickStart Bradford assay (Bio-Rad), were incubated for 80 min at 4°C with 30 μl of GTP-Sepharose bead suspension (Sigma) that was pre-treated with 100 μg/ml BSA (Pierce) at 4°C for 1 h. Samples were washed three times with 500 μl lysis buffer G, before bound protein was eluted using 100 μM GTP in lysis buffer G. The resulting eluate was added to 1× NuPAGE sample buffer (Invitrogen) and heated for 6 min at 96°C. The eluates were analyzed by SDS-PAGE and immunoblotting. Initially, protein was loaded into 4–12% (w/v) BisTris pre-cast gels (Invitrogen), prior to transfer to polyvinylidine fluoride membranes (Millipore). Non-specific bands were blocked for 1 h at 37°C with 5% (w/v) skimmed milk in PBS plus 0.1% (v/v) Tween 20. Anti-Calnexin antibody (Abcam) was used at 1:4000, and anti-FLAG antibody (Sigma-Aldrich) was used at 1:3000 at 4°C overnight. For detection, an HRP-conjugated anti-rabbit secondary antibody (Santa Cruz Biotechnology) was used at a final dilution of 1:2000, together with the SuperSignal West Pico Chemiluminescent Substrate (Pierce).

### GTPase Assay

GTPase assays were carried out according to Daniëls et al. (2011). Initially, HEK293 cells were transfected with 3× FLAG-tagged T1348N, R1398H or wild-type LRRK2 and lysed after 24 h in GTPase lysis buffer [20 mM Tris/HCl pH 7.5, 150 mM NaCl, 1 mM EDTA, 1% Triton X-100, 10% Glycerol, protease inhibitor cocktail (Roche), phosphatase inhibitor cocktail 2 (Sigma-Aldrich)]. Cell lysates were clarified as above, added to 40 μl of anti-FLAG M2 affinity gel (Sigma-Aldrich) and incubated overnight at 4°C on a turning disk in order to purify the FLAG-tagged proteins. The affinity gel was subjected to centrifugation (4°C, 100 *g*, 3 min), followed by two washes in GTPase lysis buffer and a brief rinse in GTPase buffer (20 mM Tris/HCl pH 7.5, 150 mM NaCl, 10 mM MgCl_2_, 0.02% Triton X-100). Proteins were eluted from beads with 3× FLAG peptide (Sigma-Aldrich) in GTPase buffer according to manufacturer’s instructions. The protein concentration of eluates were calculated from a serial BSA dilution curve, with purity assessed by running a small volume of each sample on an SDS-PAGE gel and staining with GelCode Blue Stain Reagent (Pierce). GTPase assays were performed according to Margalit et al. (2004) using 80 nM LRRK2 protein in GTPase buffer containing 20 U/ml pyruvate kinase (EC 2.7.1.40)/lactate dehydrogenase (EC 1.1.1.27; Sigma-Aldrich), 600 μM NADH (Sigma-Aldrich), 1 mM PEP (Sigma-Aldrich) and 500 μM GTP (Sigma-Aldrich) in a final volume of 200 μl. Reaction mixes were equilibrated to 30°C for 10 min before the reactions were initiated by the addition of GTP and thorough mixing of the contents. Depletion of NADH was measured by monitoring the decrease in absorbance at 340 nm every 5 min across a 50 min period using a VersaMax microplate reader (Molecular Devices).

### Primary Cortical Neuronal Cultures

Primary cultures of rat cortical neurones were prepared from E18 embryos obtained from timed-pregnant Sprague-Dawley rats (Taconic Biosciences, Hudson, NY, USA) narcotized with CO_2_ (in cylinders) then decapitated using a guillotine. All animal studies were approved by the National Institute of Neurological Disorders and Stroke/National Institute on Deafness and Other Communication Disorders Animal Care and Use Committee (Protocol 1151-12). Neurones were transfected with wild-type and mutant myc-tagged LRRK2 using the Amaxa Rat Neuron Nucleofector Kit, program 0-03, according to the manufacturer’s protocol (Lonza Group, Basel, Switzerland). Neurones were then plated at a density of approximately 2.6 × 10^4^/cm^2^ on cover slips and maintained as described previously (Zhu et al., 2006). At 7 days *in vitro* (DIV) neurones were fixed for 10 min with 4% paraformaldehyde, and permeabilized for 15 min with 0.05% Triton-X (Sigma-Aldrich) prior to a 1 h block in 5% NGS (GIBCO). Slides were then immunostained with primary and Alexa Fluor secondary antibodies, mounted using ProLong gold (Life Technologies), and imaged using a Zeiss LSM710 laser-scanning confocal microscope. Primary antibodies against the following proteins were used: myc-epitope (Santa Cruz Biotechnology), MAP2 (Abcam) and Tau-1 (Abcam). Alexa Fluor 488 (rabbit), 555 (mouse) and 633 (goat) secondary antibodies (Thermo Scientific) were used against myc, tau and MAP-2 epitopes respectively.

### Axon Length Measurements

Axon outgrowth properties of neurones were quantified manually. Three to six coverslips from three independent experiments for each genotype were analyzed. Axonal outgrowth and branching of dissociated neurones were quantified manually and verified using the NeuronJ plugin for ImageJ. Transfected neurones were identified using the myc-epitope antibody, whilst the length of the longest Tau-1 stained process from each neurone was measured. At least 40 neurones were quantified for each genotype, myc vector control, myc-LRRK2 wild-type, myc-LRRK2 R1398H, myc-LRRK2 R1441G, and myc-LRRK2 R1398H/R1441G from at least three coverslips.

### Luciferase Assays

Canonical Wnt activity was measured using the TOPflash reporter plasmid (Veeman et al., 2003) in human dopaminergic SH-SY5Y cells as described previously (Berwick and Harvey, 2012). Cells were extracted 24 h post-transfection using Passive Lysis Buffer (Promega) and assays performed using a Dual Luciferase Reporter Assay kit (Promega) and Turner Instruments 20/20 luminometer. Luciferase values were normalized to co-transfected Renilla plasmid to adjust for transfection efficiency, and then corrected to values from parallel experiments performed using the FOPflash control plasmid (Veeman et al., 2003).

### Statistical Analysis

GTPase assays (**Figure 4D**) were tested by two-way ANOVA with repeated measures, with the independent variables genotype, time, and time × genotype, followed by *post hoc* analysis by two-sided Dunnett’s testing. Axonal branching was assessed by Kruskal–Wallis (**Figure 6B**) or Mann–Witney (Supplementary Figure S2A) tests. Axon length was analyzed by one-way ANOVA followed by Bonferroni *post hoc* analysis (**Figure 6C**) or Student’s *t*-test (Supplementary Figure S2B). For axon length analysis, outliers (values defined as differing from the mean by 2 or more standard deviations) were first excluded. All other experiments were analyzed by one-way ANOVA for the effect of genotype followed by a two-sided Dunnett’s test with wild-type LRRK2 considered the control. Student’s *t*-test was performed using Excel, all other statistics were performed with SPSS software. All error bars represent the standard error of the mean.

## Results

### In Contrast to LRRK2 GTPase Mutants Causing PD, R1398H Increases RocCOR Dimerization

**FIGURE 2:**
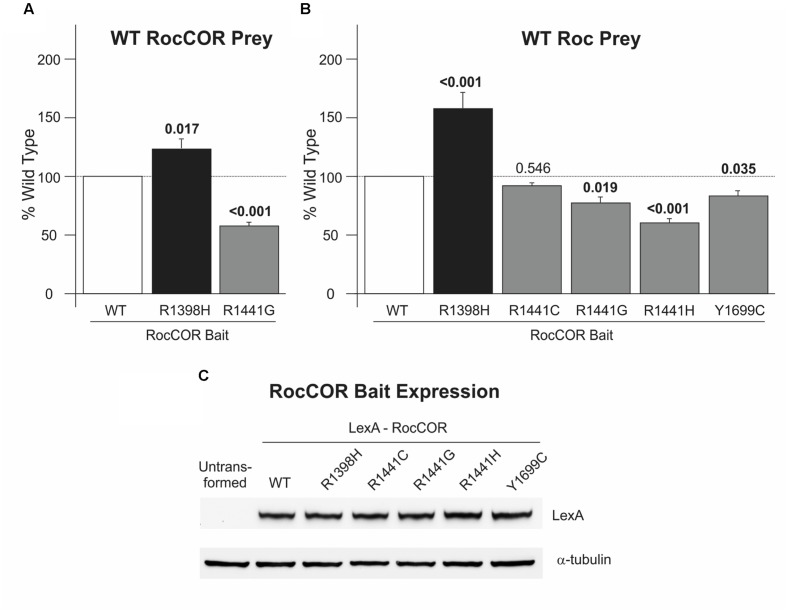
**R1398H increases LRRK2 RocCOR dimerization.** Quantitative YTH assays reveal that the presence of an R1398H variant in the RocCOR prey constructs **(A)** increases the interaction strength with a RocCOR bait whereas the pathogenic R1441G mutant has the opposite effect (one-way ANOVA for effect of genotype, *F* = 39.286, *p* < 0.001; *n* = 5). Values shown are the means of five independent experiments. **(B)** The R1398H variant also increases the interaction strength with an isolated Roc domain bait whilst the pathogenic R1441G, R1441H, and Y1699C *LRRK2* mutations weaken interaction strength (one-way ANOVA for effect of genotype, *F* = 34.729, *p* < 0.001; *n* = 3–6). **(C)** All LRRK2 mutant constructs express at an equivalent level to wild-type LRRK2. Values shown are the means of at least three independent experiments. *p*-values for *post hoc* Dunnett’s testing relative to wild-type LRRK2 are shown. Error bars represent the standard error of the mean.

To investigate functional effects of R1398H, we studied this variant in assays of LRRK2 RocCOR domain dimerization. These sensitive quantitative yeast-two hybrid (Q-YTH) assays have been used previously in our laboratory to show that the PD-causing R1441C, R1441G, R1441H, and Y1699C mutations significantly weaken RocCOR domain dimerization (Daniëls et al., 2011). Intriguingly, R1398H elicited an effect opposite to these pathogenic mutations, with increased RocCOR dimerization observed (**Figure 2A**). The R1441G mutant was studied as a pathogenic control showing as expected a significant decrease in RocCOR dimerisation (**Figure 2A**). Consistently, R1398H also strengthened interaction between the LRRK2 RocCOR tandem domain and an isolated wild-type LRRK2 Roc domain, whilst the R1441G, R1441H, and Y1699C pathogenic mutations weakened this interaction (**Figure 2B**). R1441C was also studied in this experiment, displaying a non-significant trend toward decreased interaction (**Figure 2B**). These effects were not due to changes in protein expression (**Figure 2C**). Thus, in contrast to proven PD-causing RocCOR mutations, R1398H enhanced intermolecular dimerization within the LRRK2 GTPase domain.

### Molecular Modeling Suggests that LRRK2 R1398H Affects Intramolecular RocCOR Interaction

**FIGURE 3:**
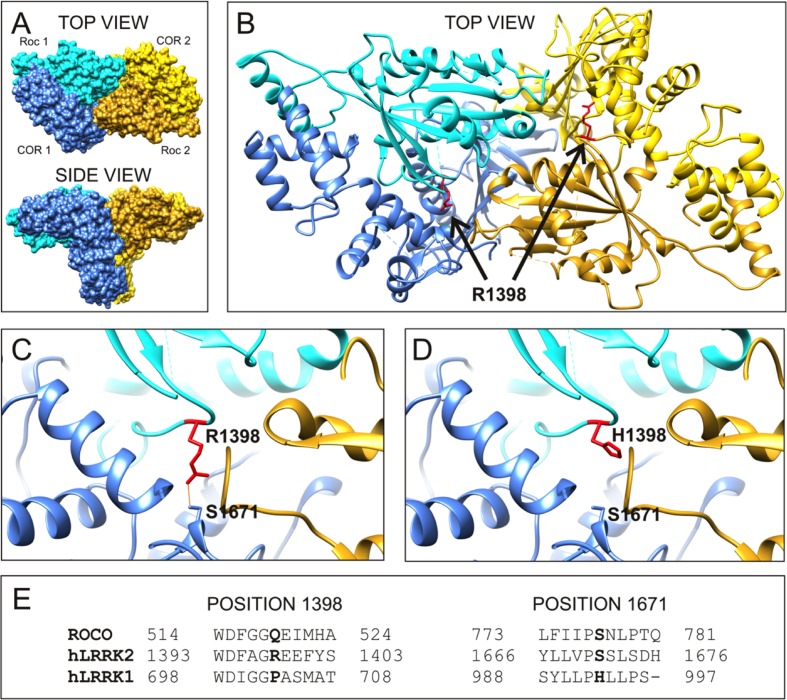
**R1398H is predicted to affect intramolecular RocCOR interactions. (A)** The topology of the dimeric RocCOR tandem domain from *C. tepidum* Roco protein is shown as a space-filled structure, viewed from two orientations. Roc and COR domains are labeled. For clarity, the Roc and COR domains of one molecule are depicted in shades of blue, the domains of the second molecule are yellow. **(B)** A magnified top view of the Roco dimer as a ribbon model. The internal location on both Roco molecules of the equivalent residue to human R1398 is colored red, and labeled. Arginine side chains are shown. **(C)** A magnified image of the modeled R1398, showing the arginine side chain projecting toward the equivalent residue S1671 in human LRRK2. A potential hydrogen bond between these residues is shown. **(D)** Swapping arginine to histidine at the R1398-equivalent site prevent hydrogen bonding to S1671. **(E)** Comparison of the sequence around R1398 and S1671 in human LRRK2 with those in human LRRK1 and *C. tepidum* Roco protein. Note that a serine at the 1671-position is conserved in mammalian LRRK2 proteins and amongst Roco proteins in lower organisms. By contrast, LRRK1 proteins contain a histidine residue at this position.

To examine the molecular mechanism underlying altered RocCOR dimerization, the predicted location of R1398 was examined by molecular modeling of the closest available protein structure: the RocCOR tandem domain of *C. tepidum* Roco protein (Gotthardt et al., 2008). Supporting the importance of R1398 in RocCOR dimerization, the equivalent residue in Roco (Q519) resides on the internal face of each Roc domain. To examine the role of human R1398, this amino acid was swapped to arginine in our model (**Figure 3C**). Using the most probable rotamer conformation, the long basic side chain of arginine projected toward the COR domain of the same molecule. Indeed, R1398 was predicted to bond with the hydroxyl group of a conserved serine in the COR domain (S778 in Roco, S1671 in LRRK2, predicted hydrogen bond distance: 2.776 Å). This is an intriguing possibility, since the R1441C/G and Y1699C pathogenic mutations have also been predicted to modify intramolecular RocCOR interactions (Gotthardt et al., 2008; Daniëls et al., 2011). In agreement, conversion of R1398 to the much shorter histidine – representative of R1398H – prevented bonding with the conserved serine (**Figure 3D**). Taken together, our molecular modeling suggests that the observed increase in *inter*molecular RocCOR dimerization caused by R1398H (**Figure 2**) likely occurs via an indirect mechanism, involving changes to *intra*molecular interactions between Roc and COR domains.

### R1398H Decreases the Fraction of GTP-Bound LRRK2

**FIGURE 4:**
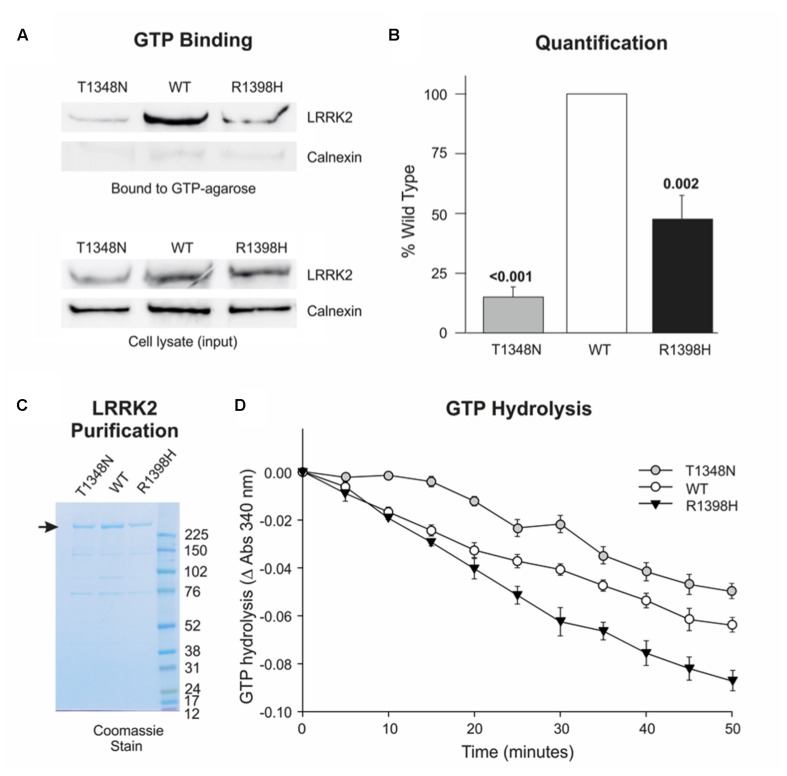
**R1398H decreases the fraction of LRRK2 in the GTP-bound ‘on’-state and increases GTPase activity. (A,B)** GTP binding assays reveal decreased binding of R1398H mutant LRRK2 to GTP-sepharose compared to wild-type. **(A)** Shows a representative experiment; **(B)** the mean of three independent experiments (one-way ANOVA for effect of genotype, *F* = 48.475, *p* < 0.001) with *p*-values from *post hoc* Dunnett’s testing indicated. Values were adjusted to the amount of transfected LRRK2 in cell lysates; gels were re-probed for calnexin to show protein loading and to confirm the purity of GTP-bound protein. **(C)** A protein gel showing the purity and concentration of FLAG-tagged LRRK2 protein to be used in GTPase assays. The masses (kDa) of the molecular weight marker bands are shown. **(D)** GTP hydrolysis assays reveal that the R1398H mutant displays greater GTPase activity than wild-type LRRK2. Two-way ANOVA with repeated measures and a Greenhouse–Geisser correction revealed significant effects of time (*F* = 224.221; *p* < 0.001), genotype (*F* = 24.06; *p* < 0.001) and interaction between time and genotype (*F* = 7.021; *p* < 0.001). *p*-values from *post hoc* two-sided Dunnett’s tests are shown. Value are the means of four independent experiments. Error bars represent the standard error of the mean.

Numerous reports indicate that PD-causing mutations in the LRRK2 RocCOR tandem domain increase the ratio of GTP-bound (‘on’) LRRK2 to GDP-bound (‘off’) LRRK2, by weakening GTPase activity and/or facilitating GTP binding (**Table 1**). In principle, a protective amino acid substitution located within this portion of LRRK2 would be expected to have the opposite effect. Thus, the effect of the R1398H variant on LRRK2 GTPase function was examined directly, using full-length human LRRK2 expressed in mammalian cells. Firstly, this variant was studied in GTP binding assays. As reported by others, wild-type LRRK2 bound strongly to immobilized GTP, whilst a well-characterized mutant, LRRK2 T1348N, had negligible GTP-binding capacity (**Figures 4A,B**). Strikingly, the R1398H variant also displayed weakened GTP binding (∼47% of wild-type), although this value was more than threefold greater than for T1348N LRRK2, indicating that GTP binding is not abolished entirely.

We next examined the effect of the LRRK2 R1398H variant on GTP hydrolysis *in vitro*, using steady-state GTPase assays. FLAG-tagged wild-type LRRK2, and LRRK2 containing T1348N and R1398H amino acid substitutions were purified from HEK293 cells (**Figure 4C**), and equimolar amounts were used in subsequent experiments (**Figure 4D**). This protein produced a steady turnover of GTP, confirming that wild-type LRRK2 possesses intrinsic GTPase activity (**Figure 4D**, open circles). Unsurprisingly, since T1348N LRRK2 is almost unable to bind GTP, this mutant possessed very little GTPase activity, with GTP hydrolysis undetectable until the 20 min time point (**Figure 4D**, gray circles). However, R1398H LRRK2 showed a marked increase in GTP hydrolysis relative to wild-type LRRK2 (**Figure 4D**, black triangles). In summary, the protective R1398H LRRK2 variant weakens GTP binding but increases steady-state GTP hydrolysis, both of which are consistent with a decrease in the proportion of GTP-bound LRRK2.

### The Protective R1398H LRRK2 Variant Increases Canonical Wnt Signaling

**FIGURE 5:**
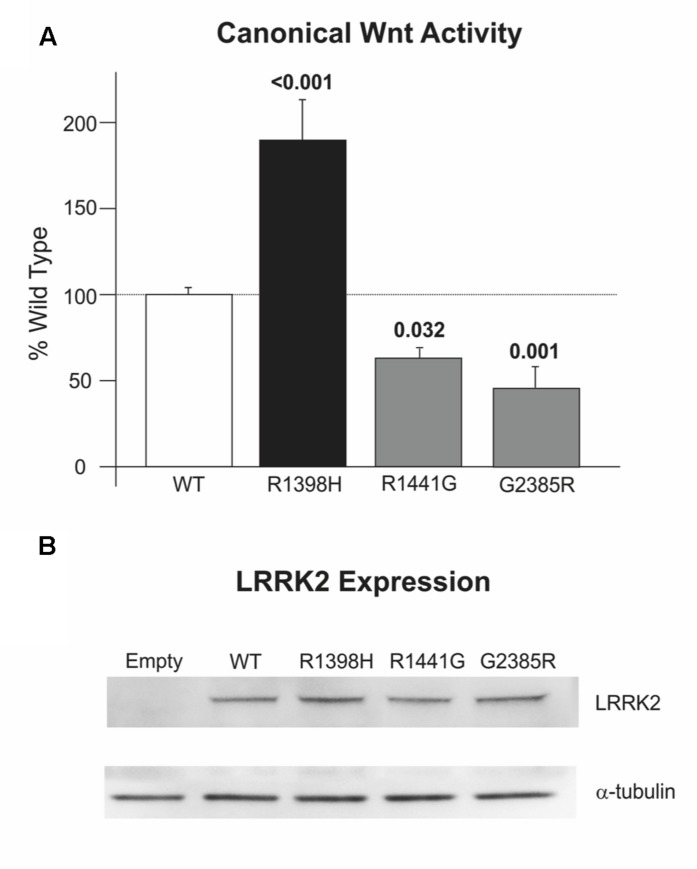
**R1398H increases canonical Wnt signaling. (A)** R1398H increases the activation of the TOPflash reporter by DVL1 relative to wild-type LRRK2, whilst the R1441G mutant and G2385R LRRK2 risk variant decreases reporter activation (one-way ANOVA for effect of genotype, *F* = 20.666, *p* < 0.001). **(B)** All LRRK2 mutant constructs express at an equivalent level to wild-type LRRK2. Values shown are the means of at least three independent experiments performed in triplicate, and are normalized to control experiments performed in parallel using the FOPflash reporter plasmid. p-values from *post hoc* two-way Dunnett’s testing are indicated (WT, *n* = 21; R1398H, *n* = 9; R1441G, *n* = 12; G2385R, *n* = 12). Error bars represent the standard error of the mean.

We have previously reported that PD-causing mutations in the Roc, COR and kinase domains of LRRK2 weaken the activation of canonical Wnt signaling that is elicited by disheveled (DVL) proteins (Berwick and Harvey, 2012). Consistent with this finding, the LRRK2 R1441G pathogenic mutant and importantly the WD40 domain G2385R PD risk variant also reduce pathway activation relative to wild-type LRRK2 in SH-SY5Y cells (**Figure 5A**). By contrast, LRRK2 R1398H enhanced DVL1-driven Wnt activation almost twice as much as wild-type LRRK2 (**Figure 5A**). This opposing effect of the protective LRRK2 variant to that described for pathogenic variants in four distinct LRRK2 domains is notable, and cannot be attributed to altered expression levels (**Figure 5B**). These experiments suggest a strong correlation between PD risk conferred by LRRK2 variants and regulation of canonical Wnt signaling activity.

To further examine a possible causal mechanism that affects canonical Wnt signaling, we investigated the interaction between the LRRK2 R1398H variant and the canonical Wnt co-receptor LRP6 in Q-YTH experiments. Using the LRP6 intracellular domain as bait and the LRRK2 RocCOR tandem domain as prey, we observed a decrease in the interaction strength with the R1398H protective variant relative to wild-type LRRK2 (Supplementary Figures S1A,B). Since pathogenic LRRK2 GTPase mutants also show a decrease in protein–protein interaction (Supplementary Figure S1A, Berwick and Harvey, 2012) altered LRRK2-LRP6 interactions do not explain the increase in canonical Wnt signaling activity of the R1398H protective variant relative to the decrease observed for pathogenic LRRK2 variants.

### Protective R1398H LRRK2 Variant Increases Axon Length in Cultured Cortical Neurones

**FIGURE 6:**
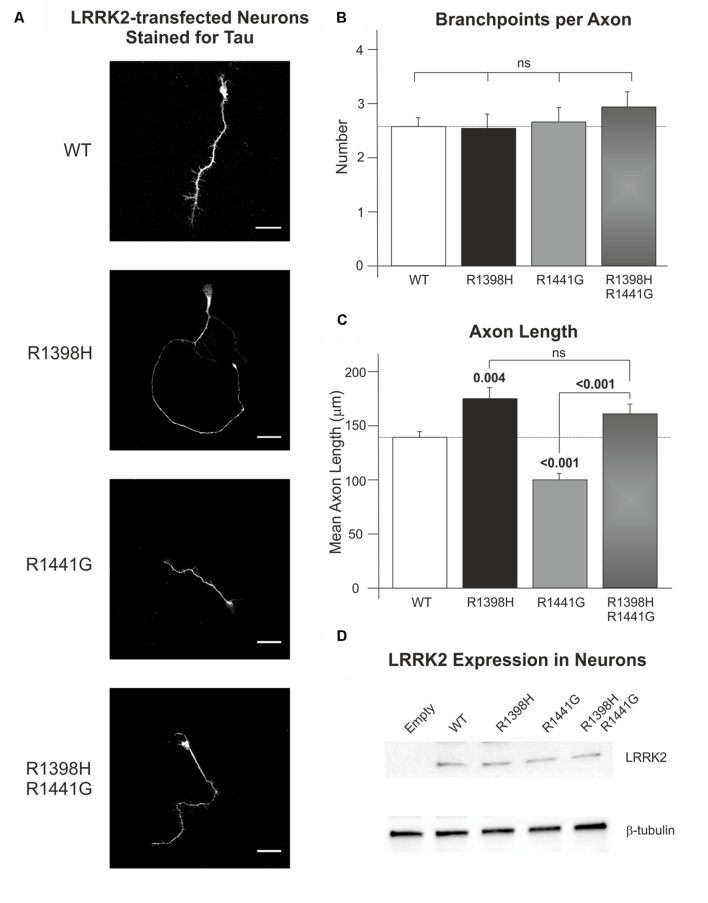
**The protective R1398H LRRK2 variant increases axonal length in primary cortical neuronal cultures.** Rat primary neurones were transfected with wild-type LRRK2 or the indicated variants prior to fixation and staining for axonal tau protein after 7 DIV. **(A)** Shows representative images of LRRK2-transfected neurones. **(B)** Quantification of the number of axon branches per neurone reveals no effect of R1441G or R1398H mutations (WT, *n* = 49; R1398H, *n* = 26; R1441G, *n* = 38; R1398H/R1441G, *n* = 31). **(C)** By contrast, R1398H increases axonal length and rescues the decrease in length caused by the pathogenic R1441G mutant (WT, *n* = 49; R1398H, *n* = 32; R1441G, *n* = 56; R1398H/R1441G, *n* = 39). **(D)** All LRRK2 mutant constructs were expressed at an equivalent level to wild-type LRRK2. Scale bar: 30 μm. *p*-values from *post hoc* Bonferroni tests are shown. Error bars represent the standard error of the mean.

Temporary differences in neurite outgrowth between LRRK2 knockout, mutant and wild-type neurones have been reported in various experimental systems (MacLeod et al., 2006; Dächsel et al., 2010; reviewed by Gómez-Suaga et al., 2014). As LRRK2 GTPase activity and Wnt signaling activity (Salinas, 2012; Gómez-Suaga et al., 2014) affect neurite outgrowth, we decided to examine axon length and branching as a correlate for neurite outgrowth and complexity in rat cortical neurones in primary culture overexpressing LRRK2 wild-type and variants at 7 DIV. Over-expression of wild-type LRRK2 had no effect on mean axonal length (*p* = 0.082) or axon branching (*p* = 0.695) relative to neurones transfected with empty vector control (Supplementary Figure S2). Furthermore, no difference in axon branching was observed between the different LRRK2 genotypes (Kruskal–Wallis χ^2^ = 0.943, *p* = 0.815; **Figure 6B**). However, the overexpression of LRRK2 mutants had a marked effect on axon length (*F* = 23.52, *p* < 0.001; **Figure 6C**). In agreement with previous work, neurones overexpressing the pathogenic LRRK2 R1441G mutant showed a reduction in axon length relative to wild-type LRRK2 (MacLeod et al., 2006; Cho et al., 2013). By contrast, the protective R1398H variant increased axon length in comparison to wild-type overexpressing cells. Remarkably, R1398H was able to rescue the effect of R1441G, as a double R1398H/R1441G mutant phenocopied the effect of the R1398H single mutant (**Figure 6**).

## Discussion

Our data provide robust experimental evidence consistent with the idea that the familial LRRK2 R1398H variant is protective against PD. Firstly, in contrast to the pathogenic R1441C, R1441G, R1441H, and Y1699C variants (**Figure 2A**; Klein et al., 2009; Daniëls et al., 2011; Law et al., 2014), R1398H increases LRRK2 RocCOR domain dimerization (**Figure 2**). Secondly, our molecular modeling suggests that this amino acid substitution will affect the interaction between the Roc and COR domains of the same molecule (**Figure 3**). Similarly, the R1441C/G/H and Y1699C mutations, and indirectly the N1437H mutation, have been suggested to affect intramolecular RocCOR interactions and consequently RocCOR dimerization as a proposed molecular pathomechanism (Gotthardt et al., 2008; Klein et al., 2009; Daniëls et al., 2011). Thirdly, our GTP-binding and GTP hydrolysis assays point toward R1398H decreasing the ratio of GTP-bound to GDP-bound LRRK2. This is in direct contrast to PD-causing mutations in the LRRK2 RocCOR tandem domain (**Table 1**). Fourthly, the R1398H protective variant increases axon length in cortical neurones, whereas LRRK2 mutants cause shortening of axons in equivalent assays (**Figure 6**, MacLeod et al., 2006; Dächsel et al., 2010; reviewed by Gómez-Suaga et al., 2014). And fifthly, R1398H has the opposite effect to pathogenic mutations and variants located *throughout* LRRK2 in cellular assays of canonical Wnt activity (**Figure 5**; Berwick and Harvey, 2012). Taken together, we believe our data make a persuasive case in support of the genetic evidence that the R1398H variant is a genuine protective variant.

Curiously, structure-based alterations at the R1398 site have already been studied in assays of LRRK2 GTPase function and produced results that are consistent with our R1398H data. This is encouraging, since mutation of R1398 to any other amino acid can be expected to prevent hydrogen bonding to S1671. The best-studied structure-based LRRK2 R1398 mutant, R1398L, was designed from Ras GTPase homology models, with the expectation that it would behave similarly to the Q61L amino acid substitution that renders Ras proteins ‘GTP-locked.’ However, R1398L had the opposite effect. This change increased GTPase activity, both in full-length LRRK2 and in a deletion construct containing the Roc, COR and kinase domains of LRRK2 (RocCOR-kinase; Xiong et al., 2010; Stafa et al., 2012; Biosa et al., 2013). R1398L also weakened GTP-binding in RocCOR-kinase constructs, although no effect was seen in full-length LRRK2 (Xiong et al., 2010; Biosa et al., 2013). Although in disagreement with expectations, these data are clearly in accordance with our results for LRRK2 R1398H. An R1398Q mutant was also studied, with the rationale that this mutation would render LRRK2 more like wild-type Ras, but no statistically significant changes to GTP-binding or GTP hydrolysis were detected using full-length LRRK2 (Biosa et al., 2013). However, when R1398Q was introduced into a RocCOR-kinase construct alongside a second Ras-like amino acid substitution, T1343G, GTP-binding was decreased and GTP hydrolysis increased (Xiong et al., 2010).

**FIGURE 7:**
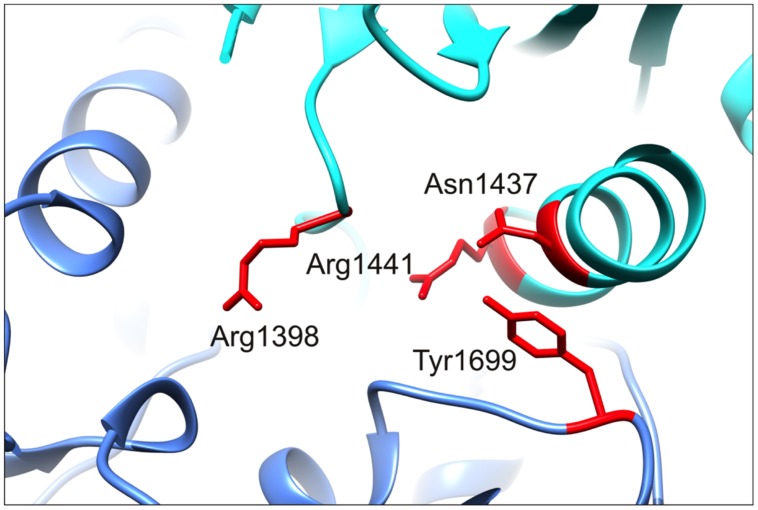
**R1398 is located in close proximity to the N1437, R1441, and Y1699 PD mutants location.** A magnified image of the *C. tepidum* Roco protein structure showing the close spatial proximity of R1398 to three sites of pathogenic mutation, N1437, R1441, and Y1699. Mutations at all four residues are expected to alter intramolecular interactions between Roc and COR domains. Note that A1442 has also been implicated as the site of a PD-causing mutation (Huang et al., 2007).

As mentioned, PD-causing mutations outside the RocCOR tandem domain do not appear to operate via the same mechanism as those affecting GTPase function, i.e., they do not shift LRRK2 GTPase toward the GTP-bound ‘on’-state. This is curious, since logic would dictate that the effects of all PD-causing mutations in *LRRK2* should converge on the same process or processes eventually. By extension, a protective variant would be expected to affect the same process, but in the opposite direction. With this in mind, it is striking that the protective LRRK2 R1398H variant appears to enhance canonical Wnt signaling. This observation is opposite to our data for pathogenic mutations throughout the catalytic core of LRRK2 (Berwick and Harvey, 2012), and a PD risk variant in the C-terminal WD40 domain (**Figure 7**).

Our data add to the growing body of evidence indicating that deregulation of canonical Wnt signaling is involved in LRRK2 PD (Berwick and Harvey, 2014). Previous studies have revealed that LRRK2 interacts with DVL proteins that play a central role in all branches of Wnt signaling: (i) canonical; (ii) planar cell polarity (PCP); and (iii) Wnt/Ca^2+^-signaling (Sancho et al., 2009; Berwick and Harvey, 2012). LRRK2 also interacts with multiple components of the β-catenin destruction complex *in vivo* and associates with the Wnt co-receptor LRP6 at membranes. Importantly, expression of familial LRRK2 mutants results in decreased activation of Wnt/β-catenin signaling (Berwick and Harvey, 2012). Strikingly, in this study we show that the R1398H mutant has the opposite effect.

Our data also have implications beyond LRRK2 PD. Wnt signaling pathways have emerged as essential regulators of neuronal development and maintenance (Inestrosa and Arenas, 2010). Wnt ligands are known to activate signaling pathways that lead to remodeling of the cytoskeleton and promote neurite outgrowth via small GTPases (Inestrosa and Arenas, 2010). Deficiencies in Wnt signaling pathways have been shown to affect synaptic stability in the striatum (Arenas, 2014), whilst antagonism of canonical Wnt signaling in the *substantia nigra* promotes dopaminergic neurone death (L’Episcopo et al., 2014). Wnt signaling is also important in the interplay between the immune system (astrocytes, microglia) and neurones, and deregulation affects adult neurogenesis (SVZ plasticity) with age (L’Episcopo et al., 2014). Furthermore, both aging and neurotoxin exposure are reported to down-regulate canonical Wnt signaling in the adult midbrain, thereby increasing the vulnerability of dopaminergic neurones. Taken together these observation provide strong evidence that Wnt signaling regulates multiple cell biological functions in the midbrain dopaminergic neurones that degenerate in PD, and that the age-related decrease in canonical Wnt activity may have a central role in the pathogenesis of sporadic forms of PD.

## Conclusion

Our data support a model where pathogenic RocCOR mutants display altered intramolecular RocCOR interactions, leading to weakened RocCOR dimerization, increased GTP binding and decreased GTP hydrolysis. Together, these effects lead to a greater proportion of LRRK2 molecules existing in the GTP-bound ‘on’-state. The protective R1398H Roc domain mutation also affects RocCOR interactions but with the opposite result, leading to more LRRK2 in the ‘off’-state. As such, developing small molecules to decrease LRRK2 GTP-binding and/or stimulate LRRK2 GTPase activity seems a promising strategy for the development of PD modifying treatments and has already shown some encouraging results in LRRK2 GTP and kinase domain mutant PD models (Li et al., 2014, 2015).

## Author Contributions

JN-A, DB, CB, and KH designed the experiments; JN-A, DB, SG, and VS performed the experiments; JN-A, DB, SG, and KH analyzed the data; DB and KH wrote the paper. All authors were involved in revising the paper for important intellectual content, and gave final approval of the version to be published.

## Conflict of Interest Statement

The authors declare that the research was conducted in the absence of any commercial or financial relationships that could be construed as a potential conflict of interest.
