# Supplementary material for: Protective LRRK2 R1398H Variant Enhances GTPase and Wnt Signaling Activity
Source: Front Mol Neurosci. 2016 Mar 8;9:18. doi: 10.3389/fnmol.2016.00018 (PMC4781896; doi:10.3389/fnmol.2016.00018)
Supplement: Supplementary file 1 [file Data_Sheet_1.PDF]

Supplementary Figures

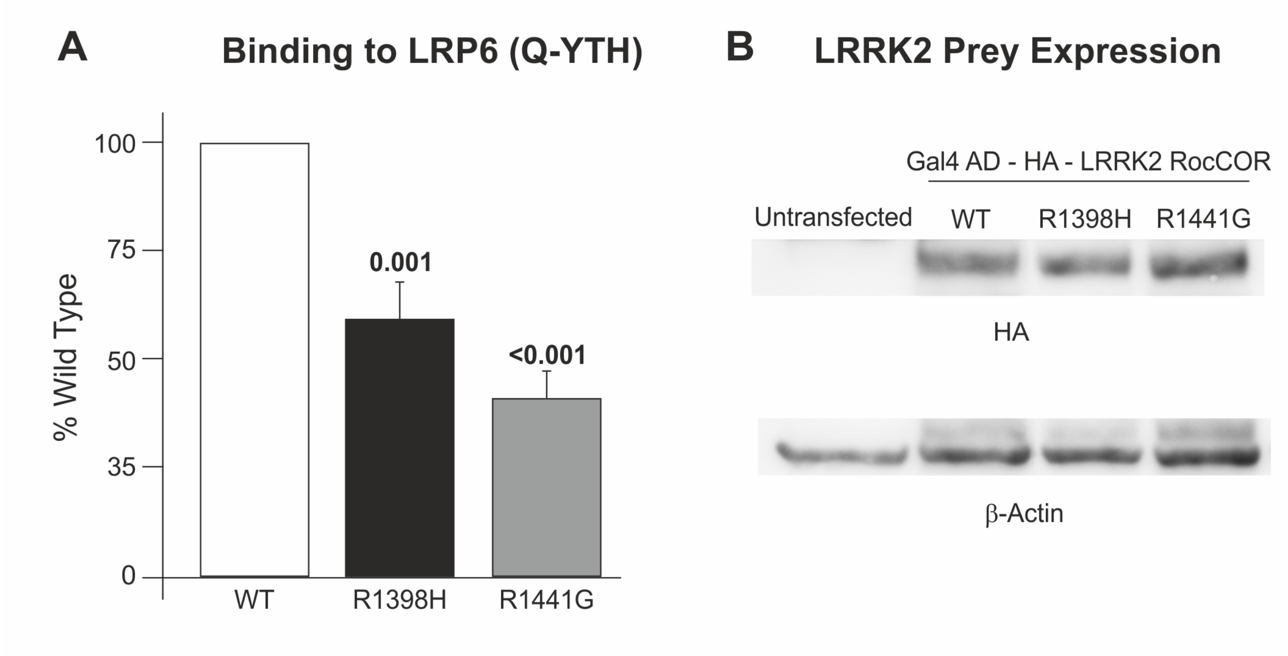

Supplementary Fig. 1 The R1398H mutant reduces LRRK2-LRP6 interaction in Q-YTH experiments. A) The R1398H and R1441G LRRK2 variants reduce the interaction with the intracellular domain of LRP6. B) All LRRK2 mutant constructs were expressed at an equivalent level to wild-type LRRK2. Values shown are the means of 5 independent experiments. 1-way ANOVA, F=22.926, $p<0.001$ for effect of genotype. p-values from post-hoc Dunnett's tests are shown.

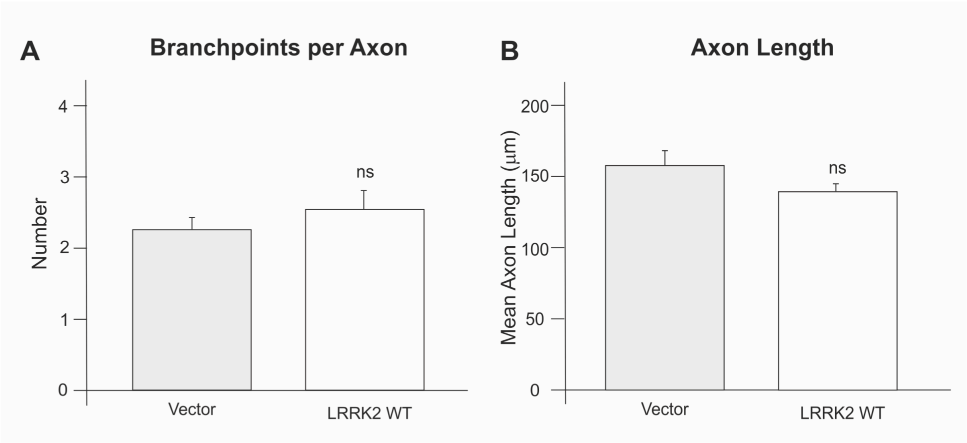

Supplementary Fig. 2 myc-LRRK2 overexpression does not affect axonal length or branch number in primary cortical rat neurons at 7 DIV. Overexpression of myc-tagged wild type LRRK2 has no effect on A) axonal branching (myc vector, n=42; myc-LRRK2, n=49) or B) axon length (myc vector, n= 41; myc-LRRK2 n=49) in comparison to overexpression of an empty vector control in primary cortical rat neurons at 7DIV.
